# Pediatric skeletal trauma - current aspects

**DOI:** 10.1007/s00068-024-02670-0

**Published:** 2024-12-10

**Authors:** Katharina Sommer, Ingo Marzi

**Affiliations:** Department of Trauma Surgery and Orthopedics, University Hospital Goethe University Frankfurt, Theodor-Stern-Kai 7, D-60590 Frankfurt / Main, Germany

This issue focuses on musculoskeletal injuries in children and adolescents, which remain a significant challenge in trauma care. The growing skeleton, with its unique age-dependent fracture patterns, excellent healing and correction potential, and associated risks for growth disturbances, requires specialized treatment and strategies distinct from adult trauma care. Current perspectives on epidemiology, diagnostics, and treatment are explored in the following papers.

Firstly, Nakao et al. [1] present a nationwide cohort study from Japan, providing comprehensive data on severe road traffic injuries in children. They highlight a decreasing trend in motorcycle crashes and in-hospital mortality over a 15-year period. However, they also report an increasing trend in severe back-seat injuries, emphasizing the need to strengthen child road safety measures, particularly concerning rear passenger seats in vehicles.

Addressing the ongoing debate about minimizing radiation exposure in pediatric trauma diagnostics, Strohm et al. [2] conducted a nationwide survey evaluating the necessity of cross-sectional imaging for fractures of the distal lower leg among German surgeons specialized in pediatric traumatology. Their results show that conventional radiographs remain the gold standard, while CT and MRI should only be used selectively, depending on the injury. This highlights the importance of adhering to ALARA (As Low As Reasonably Achievable) principles in pediatric trauma imaging and underscores the need for deeper knowledge of pediatric injuries and their specific clinical and radiological features. 

A second article in this respect by Delniotis et al., [3] examines the utility of ultrasound compared to conventional radiographs. This meta-analysis, including 17 studies, demonstrates that ultrasound has exceptionally high accuracy in detecting distal forearm fractures in children and adolescents. The use of ultrasound as a primary diagnostic tool instead of conventional radiographs can reduce radiation exposure in children. However, the findings also emphasize the necessity of standardized training and implementation of ultrasound diagnostics to achieve excellent reproducible results.

From an economic and healthcare system perspective, Spirings et al. [4] analyze a direct discharge protocol for children with torus or greenstick fractures of the distal radius or ulna in the Netherlands. They found that discharging patients with a removable orthosis and providing information through a smartphone app for patients and their parents yielded comparable outcomes to traditional treatment, which involves at least one follow-up appointment and cast immobilization of the wrist. The direct discharge protocol proved equivalent in terms of complications, functional outcomes, and patient satisfaction while reducing the burden on the healthcare system.

Regarding specific fracture patterns, Kraus et al. [5] analyze coronal shear fractures of the distal humerus in children through a multicenter study in Germany. This rare condition accounts for less than 1% of all elbow fractures in children and typically occurs around the age of 13 or older. Operative treatment is required for all displaced fractures, while only undisplaced fractures can be managed conservatively. In their study, screw fixation proved to be the preferred method, yielding good outcomes due to the fact that these fractures are mostly simple in children. 

Finally, Dietzel et al. [6] compare two minimally invasive techniques for treating unstable forearm fractures at the metaphyseal-diaphyseal junction. Both antegrade ESIN (Elastic Stable Intramedullary Nailing) and transepiphyseal K-wire fixation proved to be safe and effective. However, antegrade ESIN offered the added benefit of rehabilitation without the need for postoperative immobilization.

These contributions reflect current issues on pediatric trauma epidemiology, and in particular careful radiation protection as well as outcome-oriented treatment strategies. They provide valuable insights for optimizing care while addressing the specific needs of the growing skeleton.

## References

[1] Nakao S, Katayama Y, Kitamura T, et al. Trends and characteristics of severe road traffic injuries in children: a nationwide cohort study in Japan. Eur J Trauma Emerg Surg. 2023. 10.1007/s00068-023-02372-z 37847398 10.1007/s00068-023-02372-z PMC11666704

[2] Strohm JA, Schubert I, Schneidmüller D, et al. Is cross-sectional imaging necessary for fractures of the distal lower leg in children and adolescents: results of a nationwide survey. Eur J Trauma Emerg Surg. 2023. 10.1007/s00068-023-02379-6 37872263 10.1007/s00068-023-02379-6 PMC11666642

[3] Delniotis I, Bontinis V, Ktenidis K, et al. Diagnostic accuracy of ultrasound versus X-ray for distal forearm fractures in children and adolescents: a systematic review and meta-analysis. Eur J Trauma Emerg Surg. 2024. 10.1007/s00068-024-02451-9 38300283 10.1007/s00068-024-02451-9

[4] Spierings JF, Willinge GJA, Schuijt HJ, et al. Direct discharge for children with a greenstick or torus fracture of the wrist is a non-inferior satisfactory solution to traditional treatment. Eur J Trauma Emerg Surg. 2024. 10.1007/s00068-023-02391-w 38217672 10.1007/s00068-023-02391-w PMC11666698

[5] Kraus R, Lieber J, Schwerk P, et al. Incidence, treatment techniques, and results of distal humeral coronal shear fractures in children and adolescents—a multicenter study of the German section of Pediatric Traumatology (SKT). Eur J Trauma Emerg Surg. 2023. 10.1007/s00068-023-02370-1 37815546 10.1007/s00068-023-02370-1

[6] Dietzel M, Scherer S, Spogis J, et al. Treatment of unstable forearm fractures at the metaphyseal-diaphyseal junction in children: antegrade ESIN vs. transepiphyseal intramedullary K-wire fixation. Eur J Trauma Emerg Surg. 2024. 10.1007/s00068-024-02562-3 38819682 10.1007/s00068-024-02562-3 PMC11666750

